# Increased Bioactive Lipids Content in Human Subcutaneous and Epicardial Fat Tissue Correlates with Insulin Resistance

**DOI:** 10.1007/s11745-012-3722-x

**Published:** 2012-10-10

**Authors:** Agnieszka U. Błachnio-Zabielska, Marcin Baranowski, Tomasz Hirnle, Piotr Zabielski, Anna Lewczuk, Iwona Dmitruk, Jan Górski

**Affiliations:** 1 Department of Physiology, Medical University of Bialystok, Mickiewicza 2c, 15-222 Bialystok, Poland; 2 Department of Cardiac Surgery, Medical University of Bialystok, Bialystok, Poland

**Keywords:** Obesity, Diabetes, Ceramide, Diacylglycerols, Long-chain acyl-CoA

## Abstract

Obesity is a risk factor for metabolic diseases. Intramuscular lipid accumulation of ceramides, diacylglycerols, and long chain acyl-CoA is responsible for the induction of insulin resistance. These lipids are probably implicated in obesity-associated insulin resistance not only in skeletal muscle but also in fat tissue. Only few data are available about ceramide content in human subcutaneous adipose tissue. However, there are no data on DAG and LCACoA content in adipose tissue. The aim of our study was to measure the lipids content in human SAT and epicardial adipose tissue we sought to determine the bioactive lipids content by LC/MS/MS in fat tissue from lean non-diabetic, obese non-diabetic, and obese diabetic subjects and test whether the lipids correlate with HOMA-IR. We found, that total content of measured lipids was markedly higher in OND and OD subjects in both types of fat tissue (for all *p* < 0.001) as compared to LND group. In SAT we found positive correlation between HOMA-IR and C16:0-Cer (*r* = 0.79, *p* < 0.001) and between HOMA-IR and C16:0/18:2 DAG (*r* = 0.56, *p* < 0.001). In EAT we found a strong correlation between C16:0-CoA content and HOMA-IR (*r* = 0.73, *p* < 0.001). The study showed that in obese and obese diabetic patients, bioactive lipids content is greater in subcutaneous and epicardial fat tissue and the particular lipids content positively correlates with HOMA-IR.

## Introduction

Obesity is a complex metabolic disorder often associated with insulin resistance, type 2 diabetes, cardiovascular diseases and cancer. The molecular changes in adipose tissue that promote these disorders are not completely understood. For a long time, fat tissue was perceived as storage of free fatty acids (FFA) in the form of triacylglycerols (TAG). Over the last years, this perception of adipose tissue has been replaced by the notion that adipose tissue has a central role in lipid and glucose metabolism: expressing and secreting factors that play important endocrine functions. These factors include leptin, adiponectin, tumor necrosis factor-α (TNF-α), monocyte chemoattractant protein-1 (MCP-1), interleukin-6 (IL-6) and plasminogen activator inhibitor-1 (PAI-1) [1–3]. Over the years, studies have strengthened the notion that obesity is not a homogeneous condition and that the regional fat distribution is an important indicator for metabolic and cardiovascular alterations [4, 5].

The obesity epidemic has drawn attention to visceral adipose tissue (VAT) as a risk factor for type 2 diabetes and cardiovascular diseases. VAT has been shown to be very important source of circulating FFA. The FFA are much more easily liberated from visceral fat than from subcutaneous fat. Excess of plasma FFA leads to increased FFA uptake by tissues e.g. skeletal muscle and in the result, to intramuscular lipid accumulation. Several lines of evidence suggest that visceral adiposity rather than general adiposity might play an important role in the development of insulin resistance and cardiovascular diseases [6–8]. Epicardial adipose tissue (EAT) is a type of visceral fat that has not been studied as thoroughly as VAT and subcutaneous abdominal adipose tissue (SAT) [9]. EAT as a fat depot might function as a lipid-storing tissue–source of FFA, and as an endocrine organ secreting hormones and other biologically active molecules, which affect glucose homeostasis, energy metabolism, body weight regulation and insulin sensitivity [10–15]. It must be underlined that lipolytic activity in EAT is even higher than in other visceral fat tissues [16]. Data from several studies suggest that accumulation of intramuscular lipids is responsible for induction of insulin resistance in the tissue [17, 18]. Among these lipids are: ceramides (Cer)—central molecules in sphingolipid metabolism, diacylglycerols (DAG) and long chain acyl-CoA (LCACoA). These lipids are probably implicated not only in obesity-associated skeletal muscle but also fat tissue insulin resistance. Recent evidence suggests that adipose tissue inflammation and abnormalities in sphingolipid metabolism may contribute to the metabolic disorders associated with obesity [19]. It has been shown, that ceramide is implicated in the pathogenesis of obesity, insulin resistance [20, 21] and cardiovascular disease [22–24]. Although there is some evidence that ceramide affects insulin stimulated glucose uptake in adipose tissue in the same way as in skeletal muscle, there is very little information about biologically active lipids in SAT. We found only three reports on the ceramide content in human SAT [25–27]. Ceramide content was higher in SAT of obese men and women compared to lean non-diabetic subjects when the content of Cer was expressed per adipocyte [27]. Moreover, the ceramide level was more highly elevated in the SAT of obese woman with fatty liver than in body mass index (BMI)-matched obese individuals with no hepatic steatosis [25]. However, another report indicated that total adipose tissue ceramide content was lower in the adipose tissue from obese non-diabetic (OND) and obese diabetic (OD) subjects compared to lean non-diabetic (LND) people in spite of greater mRNA levels of SMases, SPT and CDases [26]. Another, in vitro, study demonstrated that ceramide plays an important role in the induction of insulin resistance in the adipocytes [28], but the contribution of adipose tissue to the disorder is still unclear. Nothing is known about DAG and LCACoA in adipose tissue and its implication in adipocytes as well as whole body insulin resistance. Recently, it has been found that DAG activates PKCε that results in inhibition of insulin stimulated insulin receptor kinase activity and in the result causes hepatic insulin resistance [29]. However, there is no information about the content of biologically active lipids in visceral fat. Therefore the aim of the present study was to examine the effect of obesity and type 2 diabetes on sphingolipid, DAG, and LCACoA content in human subcutaneous fat and epicardial fat tissue (as visceral fat tissue) and to understand whether relationships exist between the content of these particular lipids and insulin sensitivity.

## Materials and Methods

The study included 41 patients undergoing elective coronary bypass graft surgery. The subjects were divided into three groups: (1) lean (BMI ≤ 26.0, *n* = 14) without a history of diabetes and with normal fasting blood glucose (≤100 mg/dl) and glycated hemoglobin (≤6.5 %) level, (2) obese (BMI ≥ 30.0) without a history of diabetes and with normal fasting glycemia and glycated hemoglobin level (*n* = 12) and (3) obese with type 2 diabetes (*n* = 15). In the obese diabetic group only patients who were diagnosed as type 2 diabetic of 5–7 years duration were included in the study. All participants according to NYHA (New York Heart Association) were classified to second class that is characterized as mild symptoms and slight limitation during ordinary activity. Six of the volunteers with type 2 diabetes received insulin, two of them received metformin, one received metformin and sulfonylurea, one received insulin together with metformin, and two of the patients were on a diet. All patients were around 60 years old. We used a homeostasis model assessment for calculating insulin resistance (HOMA-IR). Blood samples were taken in a fasting state from the antecubital vein into heparinized tubes at the day of the surgery. At the beginning of the surgical intervention, a sample of epicardial adipose tissue was taken from the anterior wall of the left ventricle. Subcutaneous adipose tissue was taken from the subcutaneous fat on the sternum. Dissected tissues were promptly frozen in liquid nitrogen and then stored at −80 °C until further processing. During the surgery, blood glucose levels were kept within the physiologic range in all patients. The investigation conforms to the principles outlined in the Declaration of Helsinki and was approved by the Ethical Committee for Human Studies of the Medical University of Bialystok. All patients gave their informed consent prior to their inclusion in the study.

### Blood Samples

We measured plasma FFA concentration using UPLC/MS according to Persson et al. [30]. Briefly, the concentrations of FFA were measured against a six point standard curve constructed by taking 250 μl of the 400-μM stock solution and making dilutions with 10 mM phosphate buffer to yield 400, 200, 100, 50, 25 and 0 μM standards. Aliquots of 100 μl of plasma were taken for extraction. A quantity of 50 μl of the heptadecanoate internal standard solution was spiked to each concentration standard and each plasma sample. The standards and plasma samples were extracted with freshly prepared Dole solution composed of isopropanol:heptanes:1 M H_2_SO_4_ (40:10:1; v/v/v). The extracts were allowed to dry under nitrogen. The dried samples were resuspended in 100 μl of buffer A prior to injecting 10 μl onto the LC/MS (Agilent 6460 triple quadrupole) coupled with a Agilent 1290 Infinity UPLC system. Fatty acids were separated on the LC using a reverse-phase Zorbax SB-C18 column 2.1 × 150 mm, 1.8 μm, using two buffers. Buffer A was 80 % acetonitrile, 0.5 mM ammonium acetate; buffer B was 99 % acetonitrile, 1 % 0.5 mM ammonium acetate. The flow rate was 0.4 ml/min, and the gradient conditions were as follows: 0–3 min isocratic at 55 % B, 3–3.2 min 55–95 % B, 3.2–5 min isocratic at 95 % B, 5–5.5 min 55–95 % B, and 5.5–7 min isocratic at 55 % B.

Moreover, we measured plasma triacylglycerols, total cholesterol, HDL-cholesterol and LDL-cholesterol concentration. The plasma samples were stored at −80 °C before analysis. We also measured the fasting plasma insulin and glucose concentration for calculation HOMA-IR (homeostasis model assessment) to estimate insulin resistance.

### Adipose Tissue Bioactive Lipids

#### Sphingolipids

The content of sphingolipids was measured using a UPLC/MS/MS approach according to Blachnio-Zabielska et al. [31]. Briefly, the adipose tissue samples (40 mg) were homogenized in a solution composed of 0.25 M sucrose, 25 mM KCl, 50 mM Tris and 0.5 mM EDTA, pH 7.4. Immediately afterwards, 50 μl of the internal standard solution (17C-sphingosine and 17C-S1P, and C17-Cer Avanti polar lipids) as well as 1.5 ml of an extraction mixture (isopropanol:water:ethyl acetate, 30:10:60; v:v:v) were added to each homogenate. The mixture was vortexed, sonicated and then centrifuged for 10 min at 4,000 rpm (Sorvall Legend RT). The supernatant was transferred to a new tube and pellet was re-extracted. After centrifugation supernatants were combined and evaporated under nitrogen. The dried sample was reconstituted in 100 μl of LC Solvent A (2 mM ammonium formate, 0.15 % formic acid in methanol) for UPLC/MS/MS analysis. Sphingolipids were analyzed by means of an Agilent 6460 triple quadrupole mass spectrometer using positive ion electrospray ionization (ESI) source with multiple reaction monitoring (MRM). The chromatographic separation was performed using an Agilent 1290 infinity ultra performance liquid chromatography (UPLC). The analytical column was a reverse-phase Zorbax SB-C8 column 2.1 × 150 mm, 1.8 μm. Chromatographic separation was conducted in binary gradient using 2 mM ammonium formate, 0.15 % formic acid in methanol as solvent A and 1.5 mM ammonium formate, 0.1 % formic acid in water as solvent B at the flow rate of 0.4 ml/min.

#### Diacylglycerols

Diacylglycerols were extracted together with sphingolipids. A known amounts (50 ng) of internal standard (1,3 dipentadecanoyl-*sn*-glycerol) was added to each sample. Next, samples were extracted as described above. The following DAG were quantified: C18:1/18:2, C16:0/18:2, C16:0/16:0, C16:0/18:1, C18:0/20:0, C18:0/18:1, C18:1/18:1, C18:0/18:2 and C16:0/18:0 using UPLC/MS/MS. The chromatographic separation was performed using an Agilent 1290 infinity ultra performance liquid chromatography (UPLC). The analytical column was a reverse-phase Zorbax SB-C8 column 2.1 × 150 mm, 1.8 μm. Chromatographic separation was conducted in a binary gradient using 2 mM ammonium formate, 0.15 % formic acid in methanol as solvent A and 1.5 mM ammonium formate, 0.1 % formic acid in water as solvent B at the flow rate of 0.4 ml/min.

#### Long-Chain AcylCoA

LCACoA was measured according to Blachnio-Zabielska et al. [32]. Briefly, LCACoA was extracted with the use of ACN:2-propanol:methanol (3:1:1; v:v:v). A known amount of heptadecanoyl-CoA was added as an internal standard. The molecules (C14:0-CoA, C16:0-CoA, C16:1-CoA, C18:2-CoA, C18:1-CoA, C18:0-CoA, C20:0-CoA) were separated on a reverse-phase Zorbax SB-C18 column 2.1 × 150 mm using a binary gradient with ammonium hydroxide (NH_4_OH) in water and NH_4_OH in ACN. The LCACoA were quantified using multiple reaction monitoring (MRM) on a Triple quadrupole mass spectrometer in positive electrospray ionization (ESI) mode.

### Statistical Analysis

All data are presented as means ± SD. Data were analyzed by one-way analysis of variance (ANOVA), followed by the Newman–Keuls post hoc test. *p* values <0.05 were taken to indicate statistical significance.

## Results

### Clinical Characteristics

Clinical characteristics of the studied groups are given in Table 1. In the obese non-diabetic group and in the obese diabetic group the BMI was 37 % (*p* < 0.01) and 53 % (*p* < 0.001) higher as compared to lean non-diabetic participants. The blood glucose concentration in the obese diabetic group was about 54 % (*p* < 0.001) and 58 % (*p* < 0.001) higher compared to the lean non-diabetic and obese non-diabetic subjects respectively. The plasma insulin concentration was 62 % (*p* < 0.001) and 31 % (*p* < 0.01) higher in obese diabetic participants as compared to the lean non-diabetic and obese non-diabetic subjects respectively. There were no significant differences in the content of glucose and insulin concentration between lean non-diabetic and obese non-diabetic subjects. There were also no significant differences in total plasma cholesterol concentration between the three groups. Plasma HDL-cholesterol concentration was almost 40 % (*p* < 0.01) lower in obese diabetic group as compared to the lean individuals. Plasma concentration of the LDL-cholesterol fraction was 49 % (*p* < 0.05) and 72 % (*p* < 0.001) higher in the obese non-diabetic and the obese diabetic groups respectively as compared to the lean group. Plasma triglycerides concentration was 63 % (*p* < 0.01) and 82 % (*p* < 0.01) higher in the obese non-diabetic and obese diabetic groups, respectively compared to the lean participants. The value of glycated hemoglobin (HbA1c) was almost 29 % (*p* < 0.01) higher in the obese diabetic group as compared to the lean control group. HOMA-IR significantly increased only in the obese diabetic group (*p* < 0.001).

**Table 1:** Anthropometric and clinical parameters in the studied groups

	Control	Obese	Obese + diabetes
*n*	14	12	15
Sex (M/F)	8/6	9/3	8/7
Age (years)	64.2 ± 8.4	65 ± 8.2	66 ± 6.7
BMI (kg/m^2^)	23.7 ± 1.3	32.4 ± 4.8^b^	36.2 ± 3.8^c^
Fasting plasma glucose (mg/dl)	85.5 ± 7.7	83.2 ± 5.3	131.5 ± 12.6^c,#^
Fasting plasma insulin (μU/ml)	9.11 ± 3.1	11.3 ± 3.3	14.8 ± 2.9^c,#^
Total cholesterol (mg/dl)	157.4 ± 28.6	181.5 ± 39.7	176.5 ± 29.4
HDL-cholesterol (mg/dl)	47.3 ± 15.1	32.3 ± 7.1	28.3 ± 9.5^b^
LDL-cholesterol (mg/dl)	83.5 ± 22.1	124.2 ± 30.7^a^	143.6 ± 38.5^c^
Triglycerides (mg/dl)	119.4 ± 33	194.3 ± 67^b^	216.8 ± 69^b^
HbA1c (%)	5.9 ± 0.8	6.5 ± 0.9	7.6 ± 1.4b^
HOMA-IR	1.72 ± 0.6	2.06 ± 0.4	4.5 ± 0.9^c,^*
LVEF (%)	49.4 ± 9.6	37.3 ± 17.1	45.2 ± 15.3
LVEDd (mm)	62.3 ± 4.6	66.4 ± 12.1^a^	63.7 ± 10.1^a^
SWT (mm)	13.6 ± 3.9	13.1 ± 2.1	12.2 ± 1.8
LVPWT (mm)	11.7 ± 3.4	12.3 ± 1.9	12.6 ± 2.1
LAD (mm)	43.5 ± 4.7	48.3 ± 6.7^a^	46.4 ± 4.5

HOMA-IR (mmol/l × μU/ml) = fasting glucose (mmol/l) × fasting insulin (μU/ml)/22.5

Values are expressed as mean ± SD

*HbA1c* glycated hemoglobin, *HOMA-IR* homeostasis model assessment, *LVEF* left ventricular ejection fraction, *LVEDd* left ventricular end-diastolic diameter, *LAD* left atrial diameter, *SWT* septal wall thickness, *LVPWT* left ventricular posterior wall thickness

^a^ *p* < 0.05, ^b^ *p* < 0.01, ^c^ *p* < 0.001 versus lean non-diabetic, * p < 0.05, ^ *p* < 0.01, ^#^ *p* < 0.001 versus obese non-diabetic

### Plasma FFA Concentration (Table 2)

All the fatty acid species measured were elevated in the plasma of both obese groups. The greatest increase in both obese groups was observed in palmitate (C16:0) (~2.5 times, for both *p* < 0.001) and in stearic acid (C18:0) concentrations (by 100 %, for both *p* < 0.001) comparing to the lean non-diabetic group. The concentration of oleic acid (C18:1) increased by 63–70 % (for both *p* < 0.001) in the obese non-diabetic and obese diabetic groups respectively. The concentration of arachidonic acid (C20:4) increased by around 30 % in the obese non-diabetic group and by 48 % (*p* < 0.001) in the obese diabetic group.

**Table 2:** Plasma FFA concentration in lean non-diabetic, obese non-diabetic, and obese diabetic groups

	C14	C16:1	C16	C18:2	C18:1	C18	C20:4	Total
LND	13.3 ± 2.9	23.4 ± 2.9	89.3 ± 6.4	26.8 ± 4.8	154.6 ± 11.4	41.3 ± 2.7	18.7 ± 1.9	372.4 ± 32.5
OND	16.12 ± 3.8^a^	27.9 ± 4.1^a^	223.8 ± 36.2^c^	41.2 ± 5.8^b^	252.7 ± 37,7^c^	85.0 ± 11.9^c^	24.3 ± 2.7^c^	671.0 ± 94.5^c^
OD	16.37 ± 2.9^a^	28.3 ± 3.5^a^	238.3 ± 37.3^c^	38.3 ± 6.1^b^	263.7 ± 47.5^c^	82.5 ± 8.3^c^	27.7 ± 3.3^c^	695.2 ± 102.0^c^

Values are expressed in nmol/ml (mean ± SD)

*LND* lean non-diabetic, *OND* obese non-diabetic, *OD* obese diabetic

^a^ *p* < 0.05, ^b^ *p* < 0.01, ^c^ *p* < 0.001 versus lean non-diabetic

The linoleic acid (C18:2) concentration increased by around 54–43 % in the obese non-diabetic and obese diabetic groups, respectively (for both *p* < 0.01). The smallest, but still significant changes were noticed in myristic acid (C14:0) and palmitoleic acid (C16:1). The concentration of the fatty acids increased in both obese groups by around 20 % (for all *p* < 0.05). Total plasma FFA concentration increased by 80–87 % (for both *p* < 0.001) in the obese non-diabetic and obese diabetic groups, respectively as compared to lean non-diabetic participants.

### Fat Tissue Sphingolipids Content (Table 3)

#### Subcutaneous Adipose Tissue

The contents of SPA, S1P, C14-Cer, C16-Cer, C18:1-Cer, C18-Cer and C24:1-Cer in the subcutaneous adipose tissue were greater in obese diabetic subjects as compared to their lean non-diabetic counterparts (for all *p* < 0.001). In the obese non-diabetic group the content of SPA, C14-Cer, C24:1-Cer (for all *p* < 0.001), C18:1-Cer and C24-Cer (for both *p* < 0.05) was greater than in the lean non-diabetic group. Moreover, content of S1P, C16-Cer, C18:1-Cer and C18-Cer was markedly higher in the obese diabetic group compared to the obese non-diabetic group (for both *p* < 0.001).

**Table 3:** Sphingolipids content in white subcutaneous adipose tissue and epicardial fat tissue in lean non-diabetic, obese non-diabetic, and obese diabetic groups

	Sph	SPA	S1P	C14-Cer	C16-Cer	C18:1-Cer	C18-Cer	C20-Cer	C22-Cer	C24:1-Cer	C24-Cer	Total Cer
SubQ												
LND	0.37 ± 0.08	0.05 ± 0.01	0.028 ± 0.003	0.13 ± 0.02	2.9 ± 0.6	0.17 ± 0.02	0.18 ± 0.07	0.32 ± 0.07	1.2 ± 0.14	1.7 ± 0.3	1.2 ± 0.17	7.8 ± 0.8
OND	0.43 ± 0.08	0.11 ± 0.03^c^	0.027 ± 0.004	0.23 ± 0.06^c^	3.2 ± 1.1	0.20 ± 0.05^a^	0.21 ± 0.06	0.35 ± 0.07	1.3 ± 0.18	2.6 ± 0.3^c^	1.4 ± 0.15^a^	9.5 ± 0.9^c^
OD	0.43 ± 0.09	0.11 ± 0.03^c^	0.078 ± 0.009^c,#^	0.21 ± 0.04^c^	5.1 ± 1.3^c,#^	0.32 ± 0.07^c,#^	0.31 ± 0.05^c,#^	0.36 ± 0.11	1.3 ± 0.42	2.4 ± 0.3^c^	1.4 ± 0.42	11.4 ± 1.2^c,#^
Epicardial												
LND	0.27 ± 0.05	0.06 ± 0.01	0.025 ± 0.002	0.15 ± 0.03	3.7 ± 0.9	0.33 ± 0.08	0.49 ± 0.09	0.32 ± 0.09	0.55 ± 0.09	0.6 ± 0.1	0.5 ± 0.1	6.4 ± 1.1
OND	0.45 ± 0.09^c^	0.09 ± 0.02^c^	0.052 ± 0.006^c^	0.26 ± 0.05^c^	5.6 ± 0.5^c^	0.41 ± 0.08^a^	0.54 ± 0.09	0.33 ± 0.05	0.54 ± 0.08	0.6 ± 0.1	0.6 ± 0.1	8.9 ± 0.4^c^
OD	0.57 ± 0.11^c,^*	0.11 ± 0.03^c^	0.080 ± 0.009^c,#^	0.33 ± 0.09^c,^*	5.9 ± 0.8^c^	0.53 ± 0.08^c,#^	0.70 ± 0.09^c,#^	0.33 ± 0.06	0.60 ± 0.07	0.9 ± 0.1^c,#^	0.6 ± 0.1	9.8 ± 0.8^c,^^

Values are expressed in pmol/mg tissue (means ± SD)

*LND* lean non-diabetic, *OND* obese non-diabetic, *OD* obese diabetic. *SubQ* subcutaneous fat tissue

^a^ *p* < 0.05, ^b^ *p* < 0.01, ^c^ *p* < 0.001 versus lean non-diabetic, * *p* < 0.05, ^ *p* < 0.01, ^#^ *p* < 0.001 versus obese non-diabetic

As expected, total ceramide content was higher in both obese groups as compared to lean non-diabetic subjects (*p* < 0.001). There were also significant differences between total ceramide content in obese non-diabetic and obese diabetic group (*p* < 0.001).

#### Epicardial Adipose Tissue

In epicardial adipose tissue, the content of Sph, SPA, S1P, C14-Cer, C16-Cer, C18:1-Cer, C18-Cer and C24:1-Cer was higher in the obese diabetic group comparing to the lean non-diabetic counterpart (for all *p* < 0.001). Moreover, the content of Sph, SPA, S1P, C14-Cer, C16-Cer (for all *p* < 0.001) and C18:1-Cer (*p* < 0.05) was greater in the obese non-diabetic group than in the lean non-diabetic group. There were also differences in sphingolipid content between both obese groups. The content of Sph, C14-Cer (for both *p* < 0.05), S1P, C18:1-Cer, C18-Cer and C24:1-Cer (for all *p* < 0.001) was higher in the obese diabetic group as compared to the obese non-diabetic group. Total ceramide content was higher in both obese groups comparing to the lean non-diabetic group (for both *p* < 0.001) and was significant higher in the obese diabetic group as compare to the obese non-diabetic group (*p* < 0.01).

### Fat Tissue DAG Content (Table 4)

#### Subcutaneous Adipose Tissue

In subcutaneous fat tissue, the content of C18:1/C18:2, C16:0/18:2, C16:0/16:0, C18:0/18:1 (for all *p* < 0.001), C16:0/18:1 (*p* < 0.01) and C18:1/18:1 (*p* < 0.05) increased in the obese diabetic group as compared to lean subjects. The level of C16:0/18:2, C18:1/18:0 (for both *p* < 0.001), C16:0/18:1 (*p* < 0.01) and C18:1/18:1 (*p* < 0.05) was higher in the obese non-diabetic group as compared to the lean non-diabetic group. There were also differences in C16:0/16:0 (*p* < 0.001) and C18:1/18:2 (*p* < 0.05) content between both obese groups. The higher content of the compounds was noticed in the obese diabetic group. As expected, total DAG content increased in both obese groups as compared to the lean control group (for both *p* < 0.001).

**Table 4:** Diacylglycerols content in white subcutaneous adipose tissue and epicardial fat tissue in lean non-diabetic, obese non-diabetic, and obese diabetic groups

	C18:1/18:2	C16:0/18:2	C16:0/16:0	C16:0/18:1	C18:0/18:1	C18:1/18:1	C18:0/18:2	C16:0/18:0	Total
SubQ									
LND	13.4 ± 1.06	30.2 ± 4.5	26.9 ± 3.9	62.1 ± 10.4	2.1 ± 0.5	0.09 ± 0.01	1.65 ± 0.35	0.74 ± 0.12	137 ± 12.5
OND	14.6 ± 1.84	77.8 ± 24.4^c^	27.2 ± 2.7	76.8 ± 10.8^b^	3.2 ± 0.7^c^	0.14 ± 0.07^a^	1.78 ± 0.34	0.72 ± 0.16	202 ± 26.4^c^
OD	16.8 ± 3.06^c,^*	81.0 ± 24.0^c^	35.7 ± 5.2^c,#^	74.1 ± 9.2^b^	3.2 ± 0.6^c^	0.11 ± 0.03^a^	1.75 ± 0.30	0.68 ± 0.10	213 ± 28.6^c^
Epicardial									
LND	0.31 ± 0.05	1.58 ± 0.27	0.40 ± 0.08	2.08 ± 0.39	0.18 ± 0.04	0.03 ± 0.006	0.097 ± 0.013	0.067 ± 0.009	4.73 ± 0.54
OND	0.63 ± 0.11^c^	2.01 ± 0.35^b^	0.57 ± 0.09^c^	3.63 ± 0.74^c^	0.41 ± 0.07^c^	0.05 ± 0.007^c^	0.124 ± 0.031^b^	0.094 ± 0.019^c^	7.53 ± 0.90^c^
OD	0.66 ± 0.12^c^	2.08 ± 0.36^c^	0.68 ± 0.11^c,^*	3.61 ± 0.70^c^	0.52 ± 0.08^c,^^	0.05 ± 0.006^c^	0.118 ± 0.019^b^	0.092 ± 0.022^c^	7.82 ± 0.83^c^

Values are expressed in pmol/mg tissue (mean ± SD)

*LN-D* lean non-diabetic, *ON-D* obese non-diabetic, *OD* obese diabetic. *SubQ* subcutaneous fat tissue

^a^ *p* < 0.05, ^b^ *p* < 0.01, ^c^ *p* < 0.001 versus lean non-diabetic, * *p* < 0.05, ^ *p* < 0.01; ^#^ *p* < 0.001 versus obese non-diabetic

#### Epicardial Adipose Tissue

All measured DAG species increased in the obese diabetic group (for all *p* < 0.001, except C18:0/18:2 where *p* < 0.01) as compared to the lean non-diabetic group. Elevated content of all measured DAG (for all *p* < 0.001 except C16:0/18:2 and C18:0/18:2 where *p* < 0.01) was also noticed in the obese non-diabetic group as compared to the lean control group. Moreover, increased content of C16:0/16:0 (*p* < 0.05) and C18:0/18:1 (*p* < 0.01) was observed in the obese diabetic group comparing to the obese non-diabetic group. As expected, total DAG content in that tissue was also greater in both obese groups as compared to the lean control group (for both *p* < 0.001).

### Fat Tissue LCACoA Content (Table 5)

#### Subcutaneous Adipose Tissue

In both obese groups, the content of C16:1-CoA, C18-CoA (for both *p* < 0.05), C16-CoA and C18:1-CoA (for both *p* < 0.01), was greater as compared to the lean non-diabetic group. The total LCACoA content was also greater in both obese groups (*p* < 0.001) comparing to the lean control group. There were no statistical differences between both obese groups.

**Table 5:** LCACoA content in white subcutaneous adipose tissue and epicardial fat tissue in lean non-diabetic, obese non-diabetic, and obese diabetic groups

	C14-CoA	C16:1-CoA	C16-CoA	C18:2-COA	C18:1-CoA	C18-CoA	C20-CoA	Total
SubQ								
LND	0.021 ± 0.010	0.035 ± 0.013	0.050 ± 0.01	0.11 ± 0.02	0.30 ± 0.06	0.05 ± 0.02	0.034 ± 0.018	0.60 ± 0.09
OND	0.019 ± 0.005	0.046 ± 0.011^a^	0.068 ± 0.01^b^	0.11 ± 0.04	0.39 ± 0.10^b^	0.07 ± 0.02^a^	0.035 ± 0.009	0.74 ± 0.07^c^
OD	0.025 ± 0.020	0.045 ± 0.012^a^	0.065 ± 0.01^b^	0.12 ± 0.03	0.37 ± 0.09^b^	0.07 ± 0.02^a^	0.042 ± 0.015	0.74 ± 0.08^c^
Epicardial								
LND	0.021 ± 0.005	0.048 ± 0.01	0.06 ± 0.01	0.13 ± 0.02	0.35 ± 0.07	0.07 ± 0.02	0.046 ± 0.01	0.73 ± 0.07
OND	0.030 ± 0.009^b^	0.053 ± 0.01	0.09 ± 0.02^c^	0.13 ± 0.03	0.51 ± 0.10^c^	0.10 ± 0.03^b^	0.047 ± 0.01	0.97 ± 0.13^c^
OD	0.032 ± 0.018^a^	0.063 ± 0.01^a^	0.12 ± 0.02^c,^*	0.14 ± 0.03	0.53 ± 0.12^c^	0.11 ± 0.02^c^	0.052 ± 0.01	1.06 ± 0.13^c^

Values are expressed in pmol/mg tissue (means ± SD)

*LND* lean non-diabetic, *OND* obese non-diabetic, *OD* obese diabetic, *SubQ* subcutaneous fat tissue

^a^ *p* < 0.05, ^b^ *p* < 0.01, ^c^ *p* < 0.001 versus lean non-diabetic, * *p* < 0.05 versus obese non-diabetic

#### Epicardial Adipose Tissue

In the epicardial fat tissue, the content of C14-CoA, C16:1-CoA (for both *p* < 0.05), C16-CoA, C18:1-CoA and C18-CoA (for all *p* < 0.001) was greater in the obese diabetic group comparing to the lean control group. In the obese non-diabetic group, content of C14-CoA, C18-CoA (for both *p* < 0.01), C16-CoA and C18:1-CoA (*p* < 0.001) was higher comparing to the lean non-diabetic group. There was a difference in C16-CoA content between both obese groups. The content of C16-CoA was greater in the obese diabetic group as compared to the obese non-diabetic group (*p* < 0.001). The total LCACoA content was also higher in the both obese groups (for both *p* < 0.001) comparing to the lean non-diabetic group. There were no differences in total LCACoA content between both obese groups.

### Associations Between Bioactive Lipids and HOMA-IR (Fig. 1)

We found a strong positive correlation between the total content of ceramide in subcutaneous fat and HOMA-IR (*r* = 0.78, *p* < 0.001). The strongest correlation was noticed with C16-Cer (*r* = 0.79, *p* < 0.001). We did not find such a correlation in epicardial fat tissue. There was also a positive correlation between total DAG content in subcutaneous fat tissue and HOMA-IR (*r* = 0.64, *p* < 0.001) and between HOMA-IR and 16/18:2 DAG (*r* = 0.56, *p* < 0.001). No such associations were detected in epicardial fat tissue. In epicardial fat tissue we found a strong correlation between C16:0-CoA content and HOMA-IR (*r* = 0.73, *p* < 0.001).

**Fig. 1:**
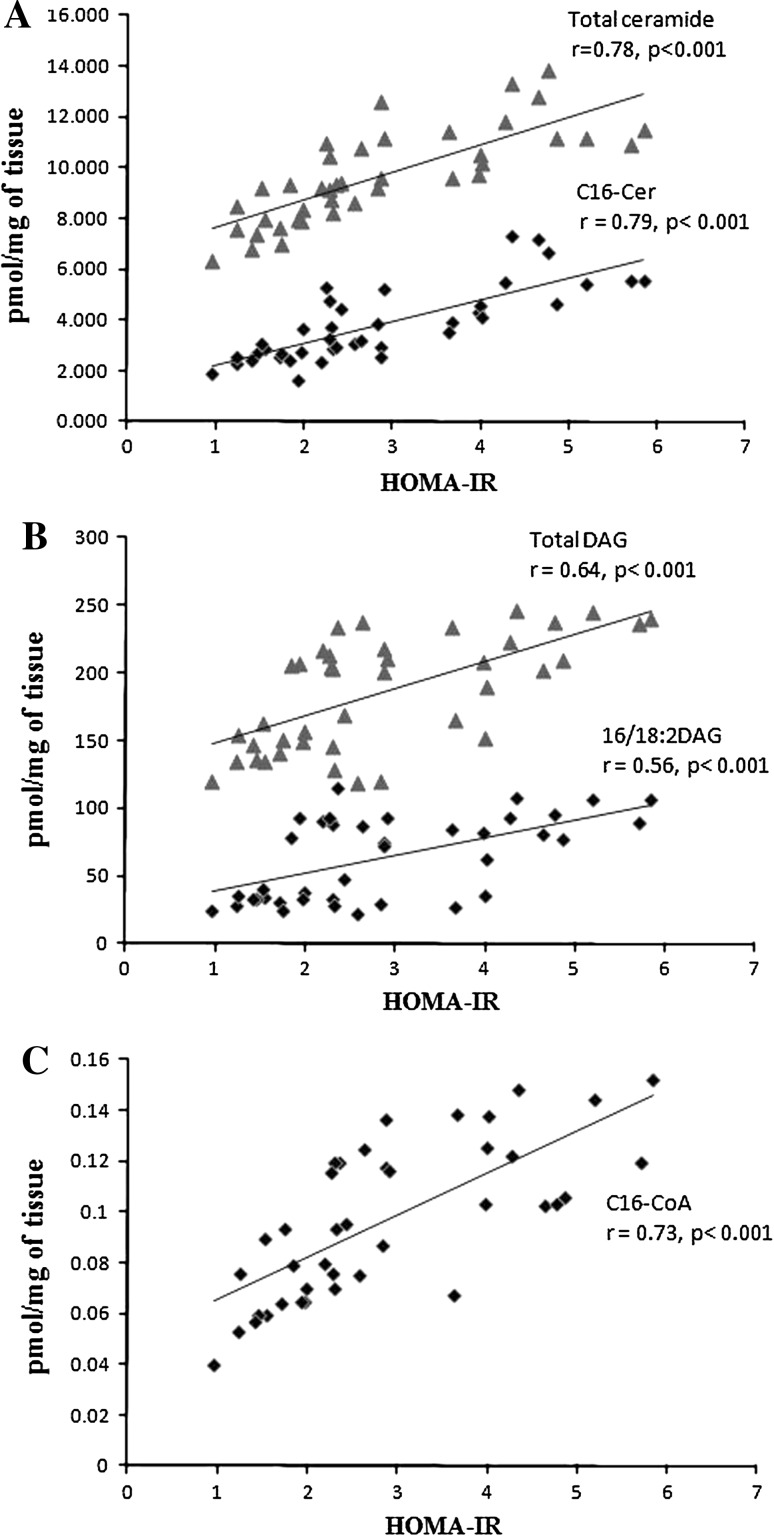
Relationship between HOMA-IR and lipids content in fat tissue. **a** *Filled triangles* correlation between total ceramide content in subcutaneous fat tissue and HOMA-IR; *diamonds* correlation between C16-Cer content in subcutaneous fat tissue and HOMA-IR, **b** *Filled triangles* correlation between total DAG content in subcutaneous fat tissue and HOMA-IR; *diamonds* correlation between C16:0/18:2 DAG content in subcutaneous fat tissue and HOMA-IR, **c** *Filled triangles* correlation between total C16-CoA content in epicardial fat tissue and HOMA-IR

### Associations Between Bioactive Lipids and Cardiac Structure Alterations

We found highly significant (*p* < 0.001 in all cases) correlations between the content of some lipid species determined in adipose tissue samples and the left ventricular end-diastolic diameter (LVEDd) of the heart. Namely, there was a positive relationship between LVEDd and the level of sphinganine, C18:1-CoA, total acyl-CoA and 18:1/18:1-DAG in the subcutaneous adipose tissue (*r* = 0.64, 0.59, 0.61 and 0.63, respectively). Moreover, a similar correlation was observed for 16:0/18:2-DAG content in the epicardial adipose tissue (*r* = 0.59). These findings suggest the existence of a link between adipose tissue lipid metabolism and alterations in cardiac structure related to the development of cardiomyopathy. However, further studies are required to reveal the nature of this relationship.

## Discussion

**Fig. 2:**
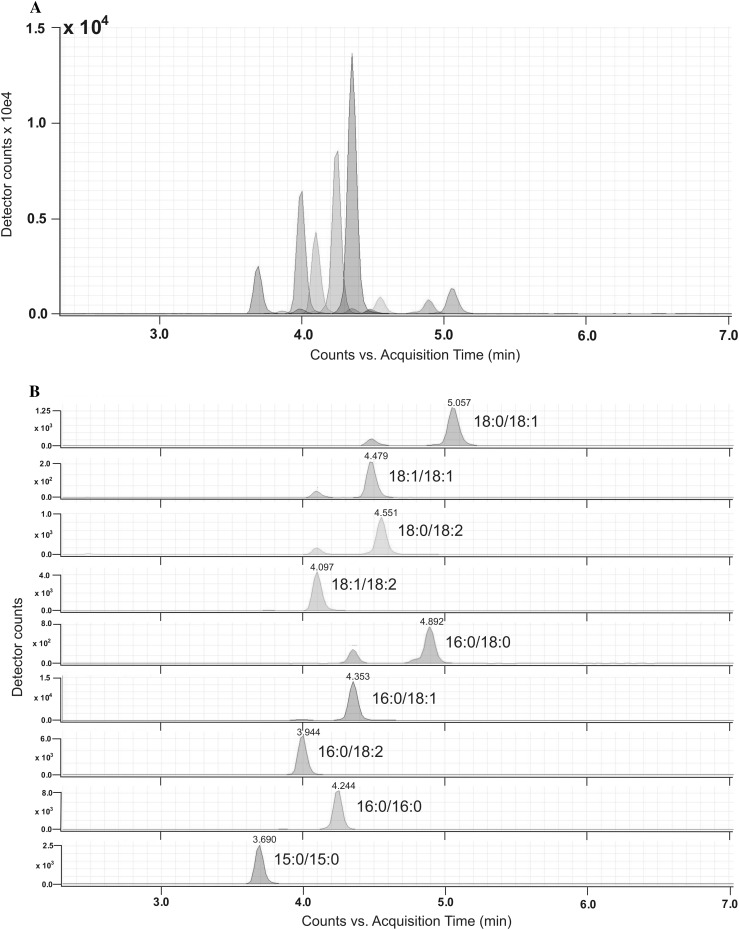
*TIC* total ion current of DAG in human subcutaneous adipose tissue (**a**). Peaks of particular DAG species (**b**)

Obesity is a major global health concern that increases the risk of metabolic and cardiovascular disease. Most of the work in the field of lipid accumulation, obesity and insulin resistance has focused on lipid metabolism in skeletal muscle [20, 33–39]. Although fat tissue is not the main tissue responsible for insulin stimulated glucose uptake, it seems to be the major tissue responsible for induction of the whole body insulin resistance. Our goal was to understand whether adipose tissues lipid content is altered in different fat tissue depots in obese non-diabetic and obese diabetic humans and, if so, whether there was any association between adipose tissue lipids and insulin resistance. In most works about obesity, fat tissue and insulin resistance, only triacylglycerols and adipokines have been taken into consideration. Ours is the first study to provide a comprehensive profile of the distinct molecular species not only of ceramides but also of DAG (TIC of DAG in SAT is presented in Fig. 2) and LCACoA within two fat depots (visceral and subcutaneous) of lean non-diabetic, obese non-diabetic and obese diabetic subjects. We have found that the lipid content is elevated in both fat tissue depots. As mentioned above, intramuscular accumulation of the lipids impairs insulin action in skeletal muscle and liver. In-vitro studies revealed that in 3T3-L1 adipocytes and in brown adipocytes, ceramide impairs insulin stimulated GLUT4 expression and glucose uptake [28]. It has also been shown that ceramide mediates the effect of TNF-α on GLUT4 mRNA content in these cells [40, 41]. Data from brown adipocytes suggest that the de novo ceramide biosynthesis plays a main role in mediating the effect of TNF-α on insulin action in these cells. There are few data from in vitro studies, showing, that pharmacological reduction of glycosphingolipids in cultured adipocytes strikingly improves glycemic control [42]. Such data prove that sphingolipids play an important role not only in regulating skeletal muscle but also adipocyte insulin sensitivity. There is some, but limited information about ceramides in human subcutaneous tissue [27]. Our previous work [26] provided information that ceramide metabolism in human subcutaneous tissue from lean healthy subjects differs from that in both obese non-diabetic and obese diabetic participants. We have demonstrated that total ceramide content decreased in subcutaneous fat tissue in both obese non-diabetic and obese diabetic patients compared to lean non-diabetic subjects. In the present work we have found, that total ceramide content as well as other measured lipids were greater in the both obese groups comparing to the lean non-diabetic group. It should be underlined, that although in both studies we used subcutaneous fat tissue, the tissue was taken from different places. In the previous study [26], the fat was taken from the abdominal region and in the present study the tissue was taken from the subcutaneous fat on the sternum. It appears that the differences in metabolic activity between fat tissues relates not only to subcutaneous and visceral but also to subcutaneous fat tissue from different regions [43, 44]. Obesity is associated with a state of chronic, low-grade inflammation and with increased plasma FFA concentrations which likely contributes to ceramide accumulation. Ceramide synthesis is activated by a variety of mediators, including proinflammatory cytokines, and an increased level of free fatty acids [45, 46]. In our work, we measured plasma FFA concentration and we found, that the concentration of plasma FFA is greater in both obese groups then in the lean group. The highest increase was found in saturated fatty acids concentrations (stearic acid and palmitic acid). There is some data showing that palmitate is responsible for lower adiponectin expression in fat tissue [47]. It is possible, that the mentioned effect of palmitate on adiponectin expression could occur through the increased level of bioactive lipids that contain palmitate.

Another key finding of our study was the positive correlation between total ceramide content in SAT and HOMA-IR (*r* = 0.78) and between C16-Cer content in subcutaneous fat tissue and HOMA-IR (*r* = 0.79) (Fig. 1a). Moreover, other positive correlations were found between HOMA-IR and total DAG content in SAT and between HOMA-IR and C16:0/18:2 (*r* = 0.56 and *r* = 0.64, respectively, Fig. 1b). We did not observe such a relation in epicardial fat tissue. However, we found a correlation between C16-CoA content in epicardial fat tissue and HOMA-IR (*r* = 0.73), (Fig. 1c). In the all cases (ceramide, DAG in subcutaneous fat tissue and LCACoA in epicardial fat tissue), the strongest correlation was found with the molecule containing palmitate. A strong positive correlation between hepatic DAG content in lipid droplets and HOMA-IR values had previously been found and that hepatic DAG content in lipid droplets is the best predictor of insulin resistance [48]. Moreover it has been postulated that increases in intracellular diacylglycerol content lead to activation of new protein kinase C (PKC) isoforms that inhibit insulin action in the liver and skeletal muscle [49].

Our data demonstrated that the biologically active lipids increase in fat tissue of obese and obese diabetic patients and correlate with insulin resistance which suggests that they might play some special role in the induction of whole body insulin resistance. However, there is still an open question as to what is the mechanism by which increased lipids content in adipose tissue affects the whole body insulin sensitivity.

In conclusion, this is the first report on bioactive lipid content in human subcutaneous and epicardial adipose tissue of lean non-diabetic, obese non-diabetic, and obese diabetic subjects. The study has shown that in obese and obese diabetic patients, bioactive lipids content is greater in subcutaneous and epicardial fat tissue and the particular lipids content correlates with insulin resistance (HOMA-IR).

## Abbreviations

ANOVA: Analysis of variance
Cer: Ceramide
CDase: Ceramidase
DAG: Diacylglycerol(s)
EAT: Epicardial adipose tissue
ESI: Electrospray ionization
FFA: Free fatty acid(s)
LCACoA: Long chain acyl coenzyme A
GLUT4: Glucose transporter type 4
HOMA-IR: Homeostasis model assessment
IL-6: Interleukin-6
LC/MS/MS: Liquid chromatography/tandem mass spectrometry
LND: Lean non-diabetic
MCP-1: Monocyte chemoattractant protein-1
OD: Obese diabetic
OND: Obese non-diabetic
PAI-1: Plasminogen activator inhibitor-1
SAT: Subcutaneous adipose tissue
SMase: Sphingomyelinase
SPT: Serine palmitoyltransferase
SPA: Sphinganine
Sph: Sphingosine
S1P: Sphingosine-1-phosphate
TNF-α: Tumor necrosis factor-α
VAT: Visceral adipose tissue
